# Incidental finding of extramammary Paget's disease during active surveillance for early‐stage prostate cancer in a prostate biopsy

**DOI:** 10.1002/iju5.12606

**Published:** 2023-07-23

**Authors:** Kengo Fujiwara, Takuma Kato, Emi Ibuki, Nachino Kimura, Yo Kaku, Hiroyuki Tsunemori, Takayoshi Miura, Teruki Dainichi, Reiji Haba, Mikio Sugimoto

**Affiliations:** ^1^ Department of Urology Kagawa University Faculty of Medicine Miki‐cho Kagawa Japan; ^2^ Departments of Diagnostic Pathology, Faculty of Medicine Kagawa University Miki‐cho Kagawa Japan; ^3^ Department of Dermatology Kagawa University Faculty of Medicine Miki‐cho Kagawa Japan

**Keywords:** biopsy, perineum, prostatic neoplasms, skin

## Abstract

**Introduction:**

Skin tissue contamination within transcutaneous visceral organ biopsies is seldom found. We encountered a rare case of extramammary Paget's disease incidentally diagnosed by prostate biopsy during active surveillance for prostate cancer.

**Case presentation:**

A 71‐year‐old Japanese patient was diagnosed with prostate cancer, and active surveillance was selected. After 1 year, prostate biopsy was performed by a transperitoneal approach, and 16 biopsy cores were taken. One biopsy core contained skin tissue showing extramammary Paget's disease. Careful skin examination confirmed the presence of an extramammary Paget's disease lesion in the left perineum, and curative surgical resection was performed. Recurrence and metastasis did not occur after 6 months of follow‐up.

**Conclusion:**

Although the perianal region is a common site of extramammary Paget's disease, early‐stage extramammary Paget's disease is often asymptomatic. Thus, during a transcutaneous biopsy, it is important to consider the appearance of the skin and the pathological features of migrating skin tissue.

**Abbreviations & Acronyms**
EMPD: extramammary Paget's disease
PSA: prostate‐specific antigen
SEER: Surveillance, Epidemiology, and End Results

**Keynote message**

Prostate biopsy via the perineum is a common method to diagnose prostate cancer. A prostate biopsy specimen occasionally contains skin tissues. In the current case, EMPD was incidentally found in migrating skin tissues in the prostate biopsy. The present case suggests that clinicians should pay attention to migrating skin tissue that can contain important information.

## Introduction

Prostate biopsy via the perineum is a standard method to diagnose prostate cancer. Although prostate biopsy specimens occasionally contain skin tissues, only the prostate tissue is considered. In the present case, EMPD was incidentally found in migrating skin tissues in the prostate biopsy.

## Case report

**Fig. 1:**
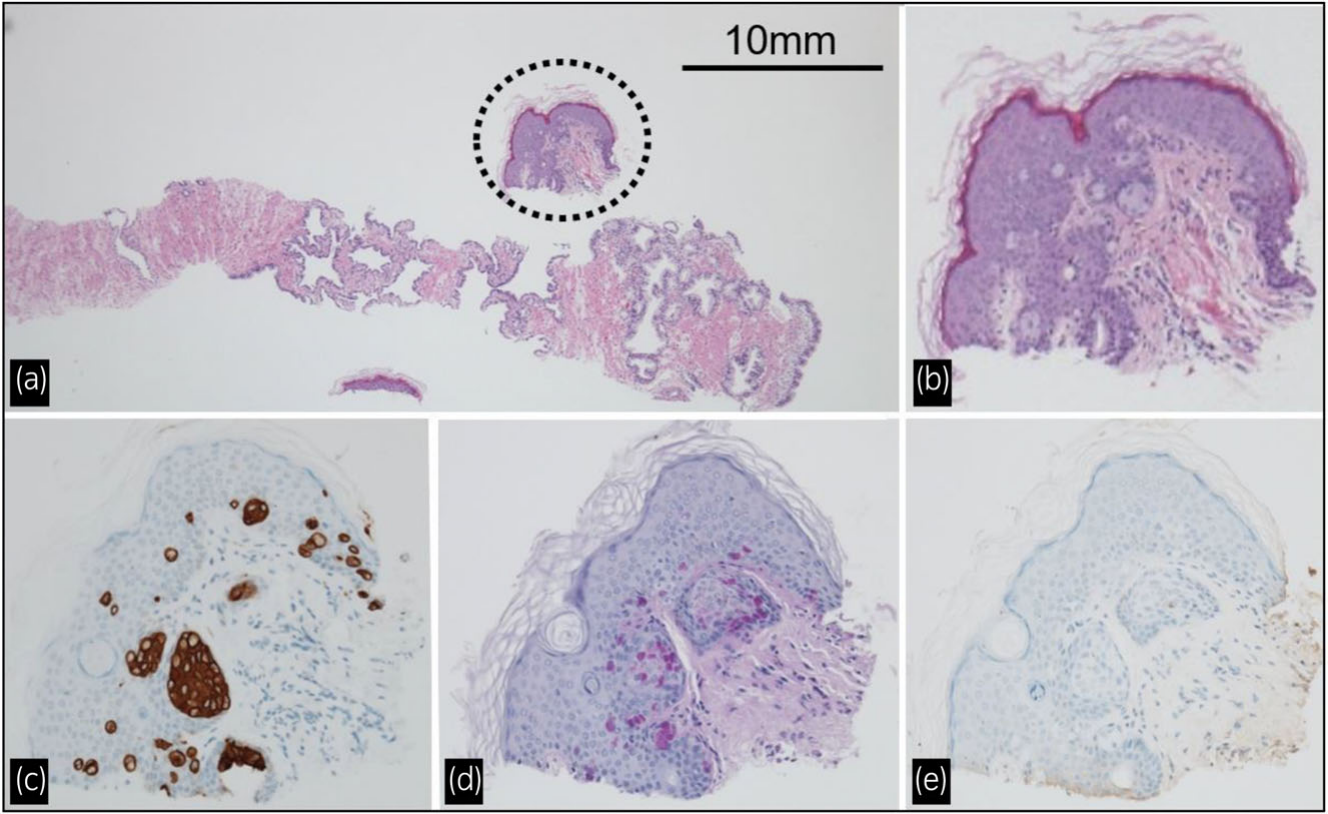
Prostate biopsy specimen. (a) A prostate biopsy specimen showing small clusters of skin tissue (dotted circle) (hematoxylin and eosin stain, ×10 magnification). (b) Atypical cells with mucus (hematoxylin and eosin stain, ×100 magnification). (c) Atypical cells stained by cytokeratin 7 (×100 magnification). (d) Atypical cells stained by periodic acid‐Schiff with diastase (×100 magnification). (e) Atypical cells unstained by PSA (×100 magnification).

**Fig. 2:**
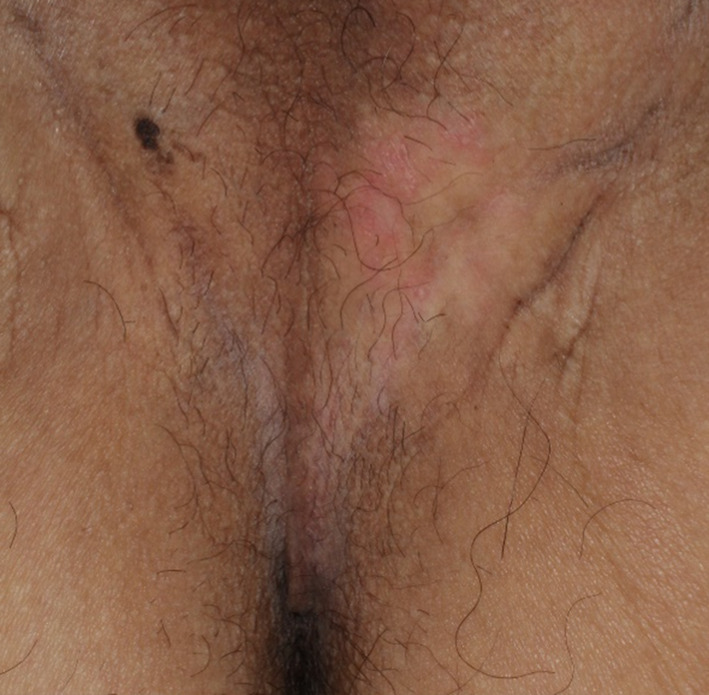
Clinical appearance of EMPD. The lesion is located midway between the anus and testicle. The surface shows a red and depigmented plaque.

A 71‐year‐old male Japanese patient visited our hospital with a chief complaint of high PSA level (8.47 ng/mL). The patient was diagnosed with prostate cancer with a Gleason score of 3 + 3 by transperineal prostate biopsy, and active surveillance was planned. The next year, the patient underwent a 16‐core transperineal repeat prostate biopsy according to the active surveillance protocol. Prostatic adenocarcinoma with a Gleason score of 6 was detected. However, one of the 16 cores contained skin tissue, and large, atypical cells with abundant pale mucinous cytoplasm in a single or nested fashion were observed within the epidermis (Fig. 1). These cells had d‐PAS‐positive intracytoplasmic mucin. Immunohistochemically, the cells were positive for cytokeratin 7 and negative for S‐100, human melanin black‐45, and PSA. EMPD was suspected, and careful examination of the patient's skin revealed a faint erythematous lesion in the left perineum. The lesion was 50 × 35 mm, slightly elevated, and it exhibited a whitish color in its periphery with a poorly circumscribed border (Fig. 2). A two‐stage surgery was planned. The primary surgery was a mapping biopsy, and the secondary surgery was radical resection. Outside of macroscopic boundaries were taken. The incision line was designed by connecting negative biopsy points. The lesion was resected at a depth of adipose tissue. Pathological examination revealed tumor cells with abundant pale cytoplasm and large nucleoli. Dermal and vascular invasion was not observed. Based on these findings, the patient was diagnosed with EMPD and pT1N0M0 stage IA. At the 6‐month follow‐up, the patient did not have signs of recurrence or metastasis.

## Discussion

The diagnostic process for this patient suggests two important clinical issues. First, early‐stage EMPD was initially undetected by both the patient and urologists. Second, prostate biopsies may contain skin tissue. The clinical course of early‐stage EMPD localized within the epidermis is indolent. However, locally invasive EMPD with a tumor thickness ≥3 mm has a 5‐year survival rate of 57%.^1^ Furthermore, distant metastasis is associated with 60% increase in mortality.^2^ Therefore, early diagnosis of EMPD is important.

In this case, neither the patient nor the urologist noticed EMPD until it was pathologically confirmed. There are several possible reasons for this finding. First, EMPD is a rare disease. Weng *et al*. analyzed the SEER database and reported a median age at EMPD diagnosis of 67.7 years.^3^ The results of a systematic review of transperineal prostate biopsies showed that the median age at biopsy in the group of prostate biopsies with antibiotics was 66 years.^4^ Although the age at EMPD diagnosis and the age at which prostate biopsy is performed are similar, EMPD is rare, with a reported incidence of 0.11/100 000 person‐years in the Netherlands population study.^5^ Therefore, even urologists, who examine male vulvodynia more than other health professionals, rarely encounter EMPD.

Second, the appearance and symptoms of EMPD are not disease‐specific. Early‐stage EMPD is characterized by multifocal, well‐circumscribed erythematous or leukoplakic plaques, or macules with occasional hyperpigmentation or hypopigmentation. While the majority of patients experience pruritus, approximately 10% of patients are asymptomatic.^2^ Because the clinical findings of EMPD overlap with those of several other diseases, the differential diagnosis for EMPD is very broad, leading to delays in diagnosis. According to Kang *et al*. and Ito *et al*., the time from symptom onset to diagnosis is 43.2 and 39.7 months, respectively.^1^, ^6^ Our patient was asymptomatic, and the urologist did not carefully investigate the skin of the perineum. For these reasons, we could not detect EMPD initially. To date, only two reports of prostate biopsies revealing an unsuspected disease exist. In one report, the unsuspected disease was urothelial carcinoma^7^; in the other, it was cryptococcal prostatitis.^8^ There are no reports of EMPD diagnosed by prostate biopsy.

We encountered a rare case of skin cancer incidentally diagnosed by transperineal prostate biopsy. During a prostate biopsy, the focus is usually on suspicious lesions in the prostate based on digital rectal examination, echocardiography, and magnetic resonance imaging findings. However, our findings suggest that the skin, which is the site of the puncture, should be carefully examined. Further, a dermatologist should be consulted, or a pathological evaluation of a skin biopsy should be performed if an abnormality is detected.

## Author contributions

Kengo Fujiwara: Writing – original draft. Takuma Kato: Writing – original draft; writing – review and editing. Emi Ibuki: Resources; writing – review and editing. Nachino Kimura: Resources; writing – review and editing. Yo Kaku: Resources; writing – review and editing. Takayoshi Miura: Resources; writing – review and editing. Hiroyuki Tsunemori: Resources; writing – review and editing. Reiji Haba: Resources; writing – review and editing. Teruki Dainichi: Resources; writing – review and editing. Mikio Sugimoto: Supervision.

## Conflict of interest

The authors declare no conflict of interest.

## Approval of the research protocol by an Institutional Reviewer Board

Our study was approved by the Kagawa University Ethics committee (approval number: 2020–204).

## Informed consent

Written informed consent was obtained from the patient for publication of this Case report and any accompanying images.

## Registry and the Registration No. of the study/trial

Not applicable.
